# MLKL Regulates Rapid Cell Death-independent HMGB1 Release in RSV Infected Airway Epithelial Cells

**DOI:** 10.3389/fcell.2022.890389

**Published:** 2022-05-31

**Authors:** Jennifer Simpson, Kirsten M. Spann, Simon Phipps

**Affiliations:** ^1^ QIMR Berghofer Medical Research Institute, Brisbane, QLD, Australia; ^2^ School of Biomedical Science, University of Queensland, Brisbane, QLD, Australia; ^3^ Centre for Immunology and Infection Control, School of Biomedical Sciences, Faculty of Health, Queensland University of Technology, Brisbane, QLD, Australia; ^4^ Australian Infectious Diseases Research Centre, Brisbane, QLD, Australia

**Keywords:** necroptosis, respiratory syncytial virus, bronchiolitis, ripk1, rage, infection

## Abstract

Respiratory syncytial virus (RSV)-induced bronchiolitis is a significant contributor to infant morbidity and mortality. Previously, we identified that necroptosis, a pro-inflammatory form of cell death mediated by receptor-interacting serine/threonine-protein kinase 1 (RIPK1) and RIPK3, and mixed lineage kinase domain like protein (MLKL), occurs in RSV-infected human airway epithelial cells (hAECs), mediating the release of the alarmin high mobility group box 1 (HMGB1). Here, we show that RSV infection of hAECs induces the biphasic release of HMGB1 at 6 (“early”) and 24 (“late”) hours post infection (hpi). The early phase of HMGB1 release at 6 hpi is cell death-independent, however, this release is nonetheless attenuated by inhibition of MLKL (primarily associated with necroptosis). The early release of HMGB1 promotes the late phase of HMGB1 release via the activation of RAGE (receptor for advanced glycation endproducts) and occurs with cell death. Treatment of hAECS with exogenous HMGB1 combined with a pan-caspase inhibitor induces hAEC necroptosis, and is attenuated by the RAGE antagonist, FPS-ZM1. Together, these findings demonstrate that RSV infection of hAECs leads to the early release of HMGB1, followed by a paracrine feed-forward amplification loop that further increases HMGB1 levels and promotes cell death. As the inhibition of MLKL or targeting of HMGB1/RAGE pathway attenuates the release of pro-inflammatory HMGB1 and decreases viral load, this suggests that the pharmacological targeting of these pathways may be of benefit for the treatment of severe RSV bronchiolitis.

## Introduction

Respiratory syncytial virus (RSV)-induced bronchiolitis is a significant contributor to infant morbidity and mortality (Nair et al., 2013). Current treatments minimize the symptoms of bronchiolitis but do little to promote disease resolution, and a childhood vaccine against RSV is yet to be approved (Meissner, 2016). Accordingly, there is a need to develop novel immunomodulatory therapies that ameliorate disease severity or limit its progression. Bronchiolitis is typically characterised by neutrophilia, oedema, epithelial sloughing, and the release of pro-inflammatory cytokines, such as the alarmin HMGB1 (Hosakote et al., 2016; Sebina and Phipps, 2020). HMGB1 is constitutively expressed in the cell nucleus, however, it can translocate to the cytoplasm and be released from the cell, whereupon it can function as a chemokine or cytokine (Janko et al., 2014). When acting as a cytokine, HMGB1 primarily elicits its effects via the activation of receptor for advanced glycation endproducts (RAGE) or toll-like receptor 4 (Yang et al., 2015; Loh et al., 2020). The extracellular release of HMGB1 can occur *via* different pathways, such as through the secretion of vesicles or following the activation of programmed cell death (Gardella et al., 2002; Yang and Tracey, 2010; Yang et al., 2015; Chen et al., 2022). Necroptosis, in particular, has been implicated in mediating the release of pro-inflammatory HMGB1 (Christofferson and Yuan, 2010; Mizumura et al., 2014; Qing et al., 2014; Simpson et al., 2020).

Necroptosis is mediated by the kinases receptor-interacting serine/threonine-protein kinase 1 (RIPK1), RIPK3, and mixed lineage kinase domain like pseudokinase (MLKL) (Christofferson and Yuan, 2010; Christofferson et al., 2012; Weber et al., 2018). Typically, RIPK1 and RIPK3 form a complex and subsequently induce the phosphorylation and oligomerization of MLKL. Phosphorylated (p)MLKL then translocates to the plasma membrane where it induces membrane damage by activating ion channels or by forming a pore in the plasma membrane to disrupt ion currents. Impaired plasma membrane integrity eventually leads to cell death and the release of alarmins such as HMGB1 (Dondelinger et al., 2014; Negroni et al., 2017). Initially, the sole function of MLKL was ascribed to necroptosis, however, it has recently been implicated in the formation of intraluminal vesicles, which become extracellular vesicles, facilitating the release of bioactive mediators such as IL-1β (Yoon et al., 2017).

Previously, we demonstrated that HMGB1 and necroptosis proteins are upregulated in two preclinical models of bronchiolitis (Simpson et al., 2017; Simpson et al., 2020) and that RSV infection of human airway epithelial cells (hAECs) induces necroptosis at 24 h post infection (hpi), leading to the release of HMGB1 (Simpson et al., 2020). Significantly, the inhibition of MLKL or RIPK1 decreased RSV-induced hAEC cell death and associated HMGB1 release *in vitro*, and conferred protection against severe bronchiolitis *in vivo* in a preclinical model (Simpson et al., 2020). However, as we had observed a small increase in HMGB1 release from the RSV infected hAECs at 12 h (Simpson et al., 2020), we sought to investigate the mechanism of this early HMGB1 release. Here we show that RSV infection induces the release of HMGB1 in a biphasic manner and that the early phase of HMGB1 release at 6 hpi is MLKL-dependent but occurs in the absence of cell death. The release of the early HMGB1 activates RAGE, initiating a second wave of pRIPK1/pRIPK3/pMLKL phosphorylation and the late HMGB1 release at 24 hpi and occurs with cell death. Therefore, the inhibition of MLKL attenuates both cell death dependent and independent release of HMGB1 from RSV infected hAECs.

## Materials and Methods

### Human Airway Epithelial Cells

Primary hAECs from healthy paediatric donors of both sexes aged 2–5 years old (commercially obtained from Lonza/Clontech Pty Ltd.) were cultured in monolayers as described previously (Simpson et al., 2020; Werder et al., 2022). Briefly, the cells were cultured in Bronchial Epithelial Cell Growth Basal Medium (BEGM CC-3171; Lonza) containing 1% hydrocortisone on chamber slides or standard 24-well cell culture plates (Thermofisher) at 37°C, 5% CO2 until 75% confluent. Hydrocortizone was removed from the BEGM 24 h prior to infection with RSV A2 strain (ATCC) at a multiplicity of infection (MOI) of 1 pfu/cell. After 2 h, the RSV inoculum was removed, and the cells washed with PBS before media was replaced. RSV A2 stocks were prepared through sucrose cushion concentration followed by high-speed pelleting of viral particles to remove the sucrose, as previously described (Spann et al., 2014). Where stated, the cells were treated with an MLKL inhibitor [necrosulfonamide (NSA) (5 µg/ml; Milipore)], pRIPK1 inhibitor [GSK’963 (1 mg/ml; GSK)], pRIPK3 inhibitor [GSK’872 (1 µg/ml; GSK)], anti-HMGB1 (5 µg/ml; R and D) or RAGE antagonist [FPS-ZM1 (0.1 µg/ml; Merck)] for 30 min prior to RSV inoculation. For specific experiments, recombinant HMGB1 (30 ng/ml; Chondrex) was added to the cells for 2 h after a 30 min incubation with pan-caspase inhibitor Z-VAD-FMK [(zVAD) (9 µg/ml; Tocris)]. Cell culture supernatant was removed at 0, 2, 4, 6, 12, 18, 24 and, 48 hpi. Cell were collected in Trizol (Ambion) for later RNA extraction or were fixed with 10% Formalin for 15 min prior to staining.

### Immunofluorescence and Quantification

The cells were stained and enumerated as previously described (Simpson et al., 2020). Briefly, fixed cells on coverslips were incubated with anti-human pMLKL (Ser358, Abcam, ab187091; 1:200 dilution), anti-human pRIPK1 (Ser166; kindly provided by GSK, 1:450), anti-human pRIPK3 (Ser227; CST, CST93654S; 1:800) anti-human RSV fusion protein (Abcam, ab20745; 1:500), anti-human cleaved caspase-3 (Asp 175) conjugated-488 (CST, CST9669; 1:200) and anti-human HMGB1 (R&D Systems, MAB1690; 1:300) overnight. Previously we demonstrated that pRIPK1 and pMLKL expression are diminished following hAEC treatment with a specific RIPK1 and MLKL inhibitor respectively, suggesting that the antibodies are specific (Simpson et al., 2020). Cells were washed 3 times in PBS/Tween-20. Cells were then incubated with the appropriate secondary antibody for 1 h at room temperature. The secondary antibodies used included goat anti-rabbit-AF555, goat anti-rabbit-AF488 or goat anti-rabbit-AF647, goat anti-mouse-AF488, donkey anti-goat-AF555 or goat anti-rat-AF647 (all Sigma; 1:500). Nuclear stain was achieved with use of DAPI (Sigma; 1:10,000) for 5 min at room temperature. Slides were mounted using anti-fade fluorescent mounting media (Dako) and imaged on Zeiss 780-NLO Point scanning confocal or Zeiss AxioScop2. For propidium iodide (PI) (Thermofisher; 1:1,000) staining, the live cells were incubated with PI for 10 min at room temperature before washing with distilled water and mounting as above. For Sytox green (Thermofisher; 1:5,000) staining, live cells were incubated for 20 min before washing and fixing cells as above.

Immunoreactivity was enumerated by quantifying the number of pRIPK1, pRIPK3, pMLKL and cleaved caspase-3 positive cells as a percentage of total cells. For quantification of HMGB1 immunoreactivity, the nucleus and cytoplasm were individually counted and expressed as a percentage of total cells. Specifically, cells with any HMGB1 staining in the cytoplasm were classified as cyto-HMGB1 positive and cells with nuclear HMGB1 staining were classified as nuc-HMGB1 positive. All cells in one or two visual fields were counted for each donor for each antibody stain at each time point or experimental condition. Each visual field measured 400 µM × 400 µM and the average cell counts for each visual field ranged between 40 and 100 cells. Arbitrary group numbers were assigned to each group and experiment to allow for blinded quantification.

### Detection of High Mobility Group Box 1 and dsDNA

HMGB1 concentration in the cell culture supernatants was quantified by ELISA according to the manufacturer’s protocol (Chondrex, Redmond, WA, US). dsDNA was quantified by Quant-iT PicoGreen dsDNA Assay Kit according to the manufacturer’s protocol (ThermoFisher).

### Quantitative Real Time PCR

hAECs were incubated in TriReagent solution (Ambion) and a phenol-chloroform extraction performed to obtain RNA. DNAse digestion was performed with Turbo DNAse (Ambion), and reverse transcription using M-MLV reverse transcriptase and random primers (Invitrogen). qPCR was performed with SYBR Green (Life Technologies) and primers for HMGB1 and RSV. The primer sequences: HMGB1 forward, AGG​ATC​TCC​TTT​GCC​CAT​GT; HMGB1 reverse, TGA​GCT​CCA​TAG​AGA​CAG​CG; RSV forward, AAG​GGA​TTT​TTG​CAG​GAT​TGT​TT; RSV reverse, CTC​CCC​ACC​GTA​GCA​TTA​CTT​G. Expression values were normalized relative to the housekeeping gene β-actin and expressed as fold change relative to naïve (vehicle treated) cells using the 2^−ΔΔCT^ formula.

### Statistical Analysis

**FIGURE 1:**
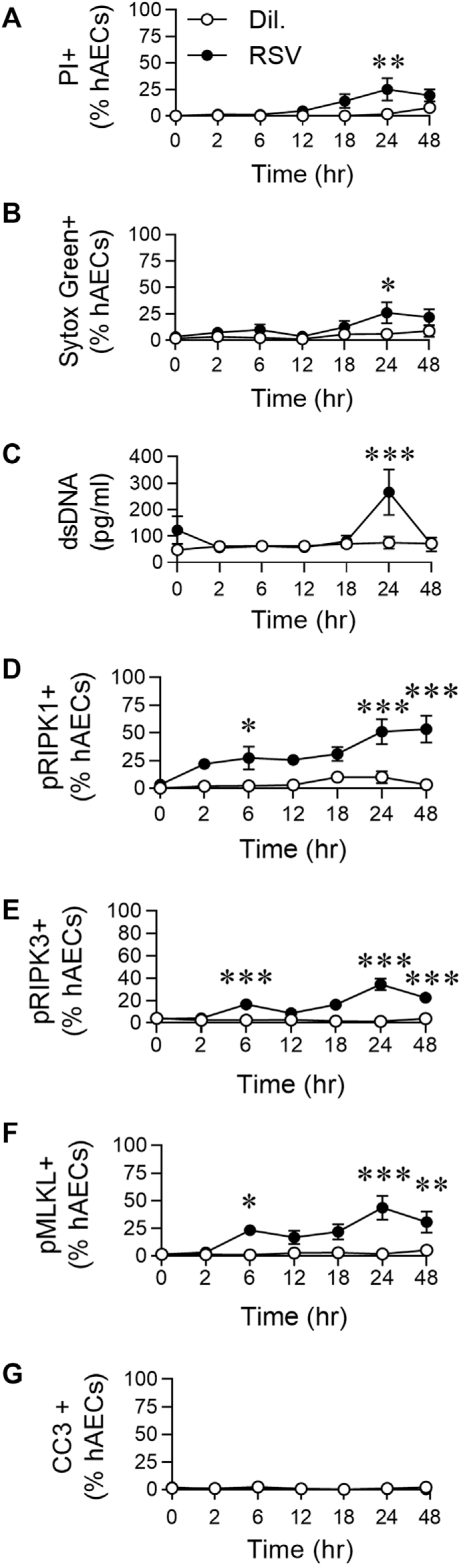
RSV infection of hAECs induces phosphorylation of necroptosis proteins prior to the induction of cell death. hAECs were inoculated with RSV (MOI of 1) and the cells analysed at 0, 2, 6, 12, 18, 24, and 48 h post infection (hpi). **(A)** Quantification of propidium iodide (PI) positive hAECs. **(B)** Quantification of Sytox Green positive hAECs. **(C)** dsDNA levels in the cell culture supernatant. **(D–G)** Quantification of **(D)** pRIPK1 positive hAECs, **(E)** pRIPK3 positive hAECs, **(F)** pMLKL positive hAECs, **(G)** cleaved caspase-3 (CC3) positive hAECs. Each data point represents 4-6 donors. The data are presented as mean ± SEM and were analyzed using a two-way ANOVA and Sidhak’s multiple comparisons test. *p < 0.05, **p < 0.01, ***p < 0.005, denotes significance between infected and uninfected cells at the specified time point.

**FIGURE 2:**
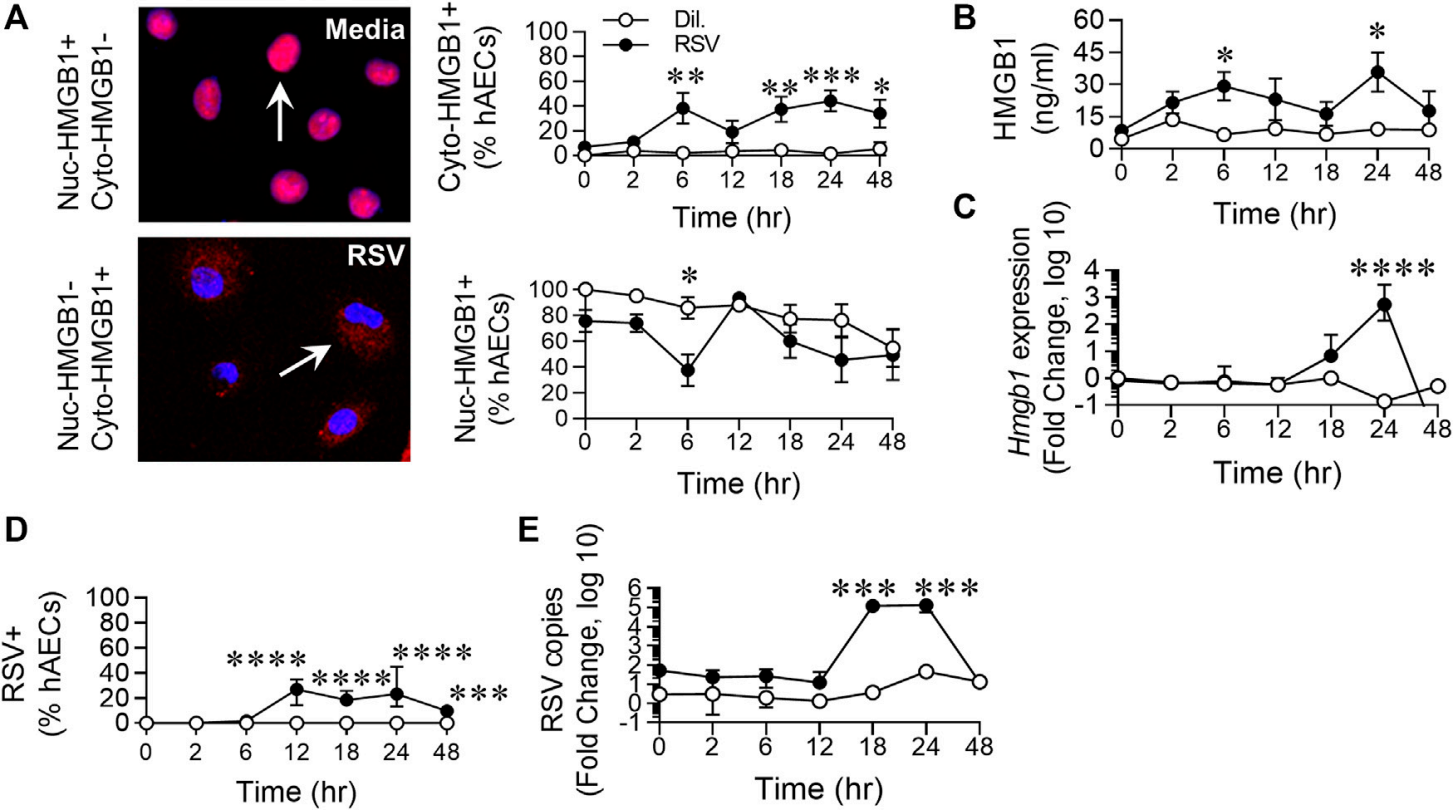
RSV infection of hAECs induces the biphasic release of HMGB1. hAECs were inoculated with RSV (MOI of 1) and the cells analysed at 0, 2, 6, 12, 18, 24, and 48 h post infection (hpi). **(A)** Quantification and representative images (magnification ×20) of nuclear- and cytoplasmic-HMGB1 positive hAECs. **(B)** HMGB1 levels in the cell culture supernatant. **(C)** HMGB1 gene expression normalized to the house-keeping gene. **(D)** Quantification of RSV positive hAECs. **(E)** Number of viral copies (expression of RSV N gene) normalized to a house-keeping gene. Each data point represents 4-6 donors. The data are presented as the mean ± SEM. The data were analyzed using a two-way ANOVA and Sidak’s multiple comparisons test. *p < 0.05, **p < 0.01, ***p < 0.005, denotes significance between infected and uninfected cells at the specified time point.

**FIGURE 3:**
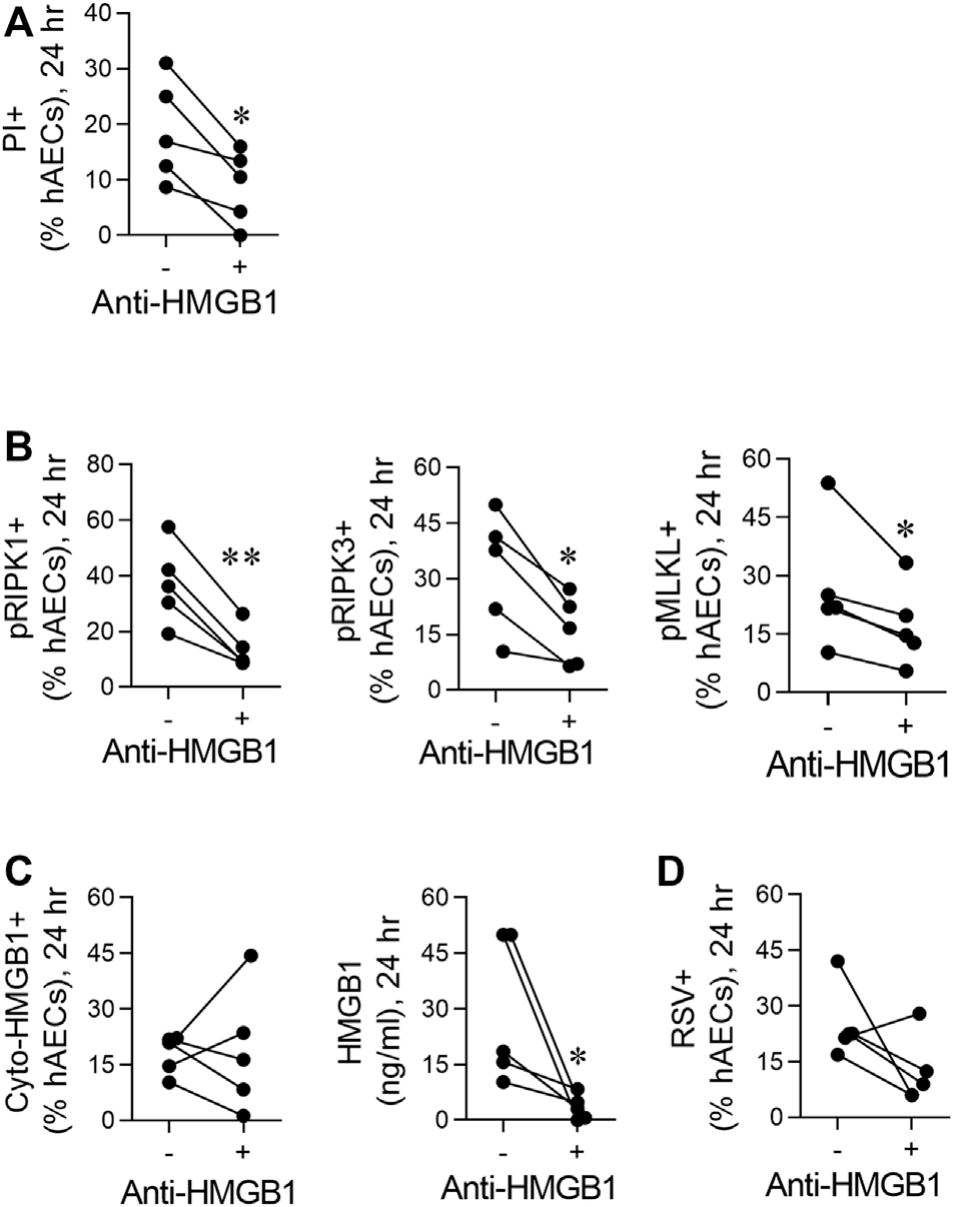
RSV induces necroptosis in hAECs in an HMGB1-dependent manner. hAECs were pre-treated with a neutralising HMGB1 antibody then inoculated with RSV (MOI of 1). Endpoints were assessed at 24 hpi. **(A)** Quantification of PI positive hAECs. **(B)** Quantification of pRIP1K, pRIPK3 and pMLKL positive hAECs. **(C)** Quantification of cytoplasmic HMGB1 and HMGB1 levels in the cell culture supernatant. **(D)** Quantification of RSV positive hAECs. Data are presented as dot plots with each data point representing one of 5 donors. The data were analyzed using a paired T-test *p < 0.05, **p < 0.01 denotes significance between anti-HMGB1 treated and untreated cells.

**FIGURE 4:**
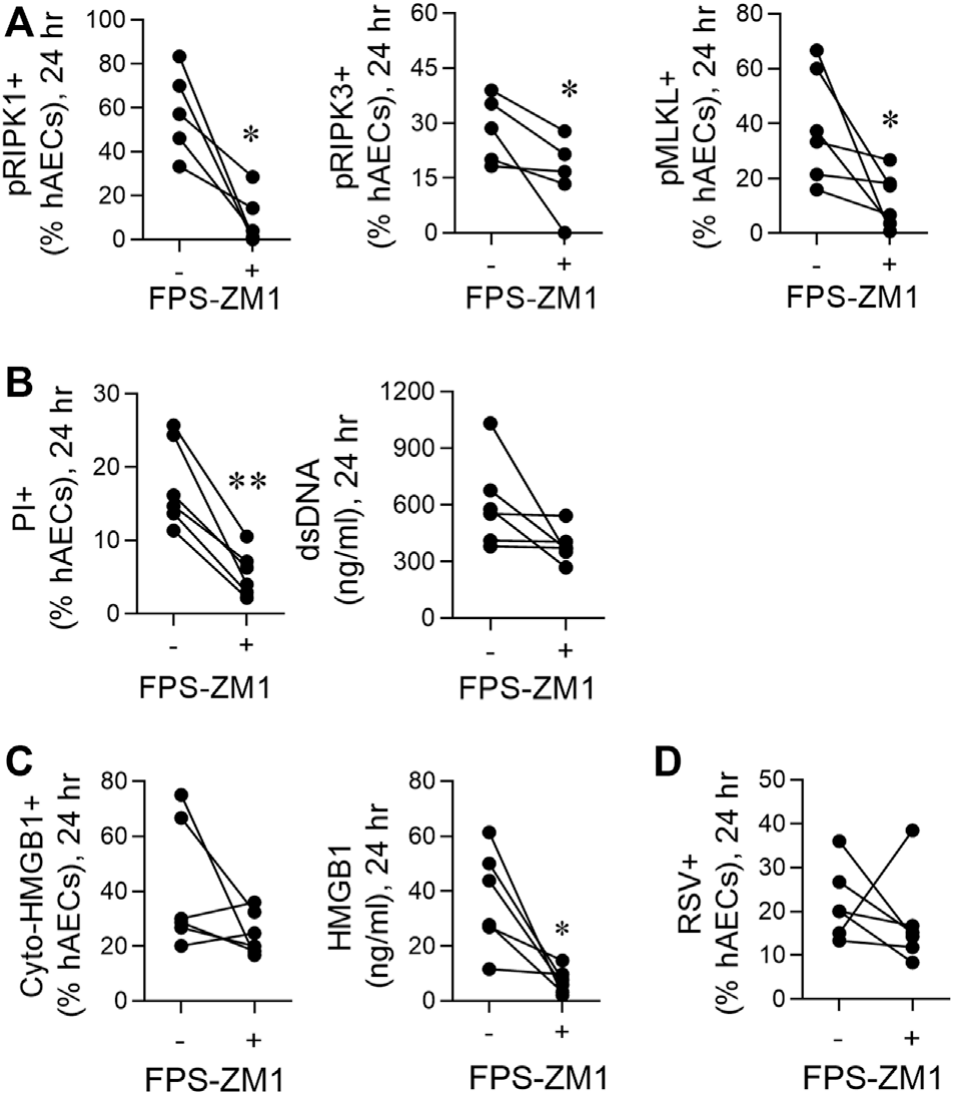
RSV-induced necroptosis is mediated via a HMGB1/RAGE axis. hAECs were pre-treated with or without a RAGE antagonist (FPS-ZM1) and infected with RSV (MOI of 1). Endpoints were assessed at 24 h post infection. **(A)** Quantification of pRIP1K, pRIPK3 and pMLKL positive hAECs. **(B)** Quantification of PI positive hAECs and dsDNA levels in the supernatant. **(C)** Quantification of cytoplasmic HMGB1 positive hAECs and HMGB1 protein expression in the supernatant. **(D)** Quantification of RSV positive cells. Data are presented as dot plots with each data point represents one of 6 donors. Data were analyzed using a paired T-test. *p < 0.05, **p < 0.01, denotes significance between RSV-infected cells treated with or without RAGE antagonist.

**FIGURE 5:**
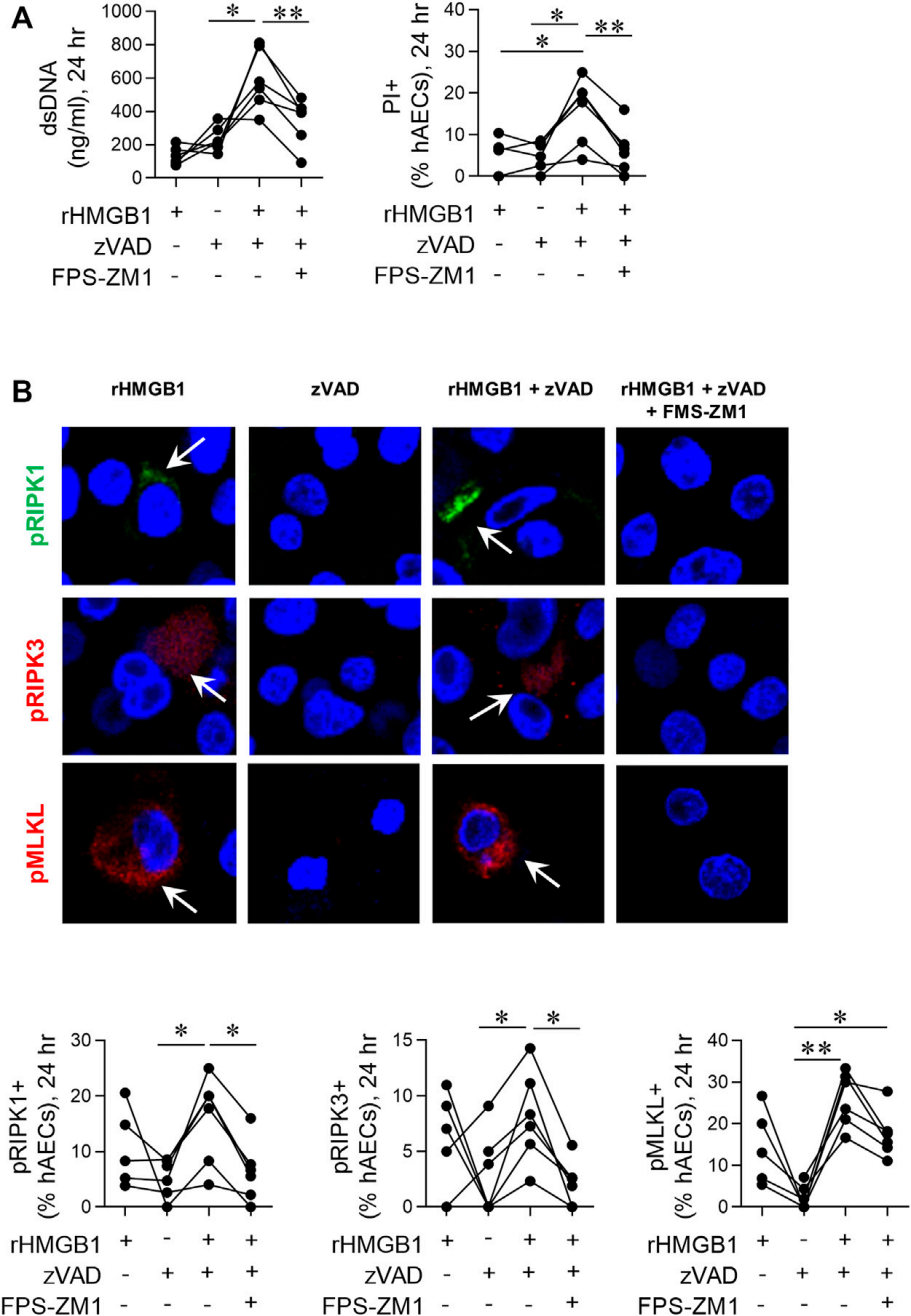
Exogenous HMGB1 and caspase inhibition induces necroptosis in hAECs. hAECs were pre-treated with a RAGE antagonist, zVAD and/or disulphide HMGB1 as indicated. Endpoints were assessed at 24 hpi. **(A)** dsDNA levels in the supernatant and quantification of PI positive hAECs. **(B)** Representative images (magnification ×40) and quantification of pRIP1K, pRIPK3 and pMLKL positive hAECs. Data are presented as dot plots with each data point representing one of 5 or 6 donors. Data are analyzed as a one-way ANOVA or mixed effects analysis and Sidhak’s multiple comparisons test. *p < 0.05, **p < 0.01, denotes significance between different treatment groups.

**FIGURE 6:**
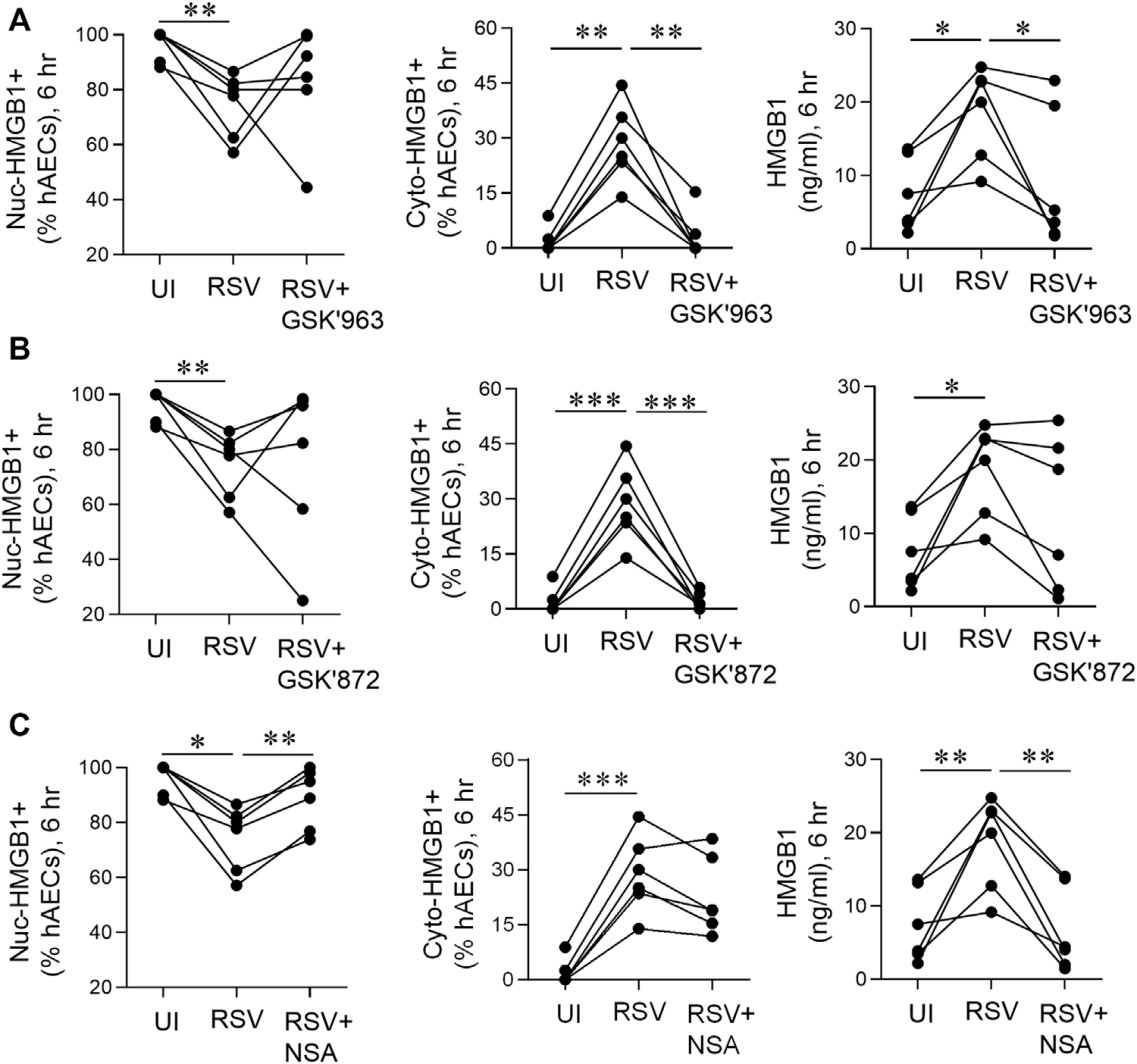
pMLKL inhibition decreases early HMGB1 release induced by RSV. hAECs were pre-treated with or without a pRIPK1, pRIPK3 or pMLKL inhibitor and infected with RSV (MOI of 1). Endpoints were assessed 6 hpi. **(A)** Quantification of nuclear and cytoplasmic HMGB1 positive hAECs and HMGB1 protein expression in the supernatant after pRIPK1 inhibition. **(B)** Quantification of nuclear and cytoplasmic HMGB1 positive hAECs and HMGB1 protein expression in the supernatant after pRIPK3 inhibition. **(C)** Quantification of nuclear and cytoplasmic HMGB1 positive hAECs and HMGB1 protein expression in the supernatant after pMLKL inhibition. The same data in the UI and RSV groups are used repeatedly across panels **(A–C)**. Data are presented as dot plots with each data point representing one of 6 donors. Data were analyzed using a one-way ANOVA and Sidhak’s multiple comparisons test. *p < 0.05, **p < 0.01, ***p < 0.005, denotes significance between different treatment groups.

Time course data is presented as mean and SEM (Figures 1, 2 and Supplementary Figure S1) and analyzed by two-way ANOVA and Sidak’s multiple comparisons test. All other data (Figures 3–6; Supplementary Figure S2) are presented as individual data points representing an individual hAEC donor and analyzed by paired Student T test or one-way ANOVA with Sidak’s multiple comparisons test. The software package GraphPad Prism 6.01 (GraphPad Software, San Diego, CA) was used for all data analysis and preparation of graphs.

## Results

### Respiratory Syncytial Virus Infection of Human Airway Epithelial Cells Induces Phosphorylation of Necroptosis Proteins Prior to the Induction of Cell Death

We previously demonstrated that RSV infection of hAECs leads to a significant increase in pRIPK1/pMLKL expression and HMGB1 release at 24 hpi (Simpson et al., 2020). Here, to determine whether pRIPK1, pRIPK3, pMLKL and HMGB1 expression were elevated at earlier time points, we performed a time course, sampling at 2, 6, 12, 18, 24 and, 48 hpi. Indicators of cell death, including propidium iodide (PI) and Sytox green positivity, as well as dsDNA release, were not significantly elevated compared to uninfected controls before 24 hpi (Figures 1A–C). Consistent with our previous observations (Simpson et al., 2020), the cell death at 24 hpi coincided with a significant increase in the expression of pRIPK1, pRIPK3 and pMLKL (determined by immunofluorescence and expressed as a percentage of AECs), but not active caspase-3 (Figures 1D–G; Supplementary Figure 1SA). However, the expression of all three phosphorylated kinases was elevated compared to uninfected controls as early as 6 hpi, before declining at 12 hpi and then increasing at 24 hpi, indicating a biphasic response (Figures Figure1D–F) in which the early phosphorylation event is independent of cell death. Indeed, of the pMLKL + cells at 6 hpi (∼20% of all hAECs), less than a fifth of these cells were also Sytox Green+, whereas at 24 hpi, the vast majority of pMLKL + cells were Sytox Green + (Supplementary Figure S1B). In summary, RSV infection induced an “early” (6 hpi) and “late” (24 hpi) phosphorylation of RIPK1/RIPK3/MLKL, however, necroptotic cell death was restricted to the late phase.

### Respiratory Syncytial Virus Infection of Human Airway Epithelial Cells Induces the Biphasic Release of High Mobility Group Box 1

Similar to the phosphorylation of the necroptosis-associated kinases, RSV infection induced an increase in cytoplasmic (cyto-)HMGB1 levels at 6 hpi, compared to the control, followed by a late increase in cytoplasmic HMGB1 from 18 hpi. The increased cyto-HMGB1 at 6 hpi coincided with a decrease in nuclear HMGB1, which also appeared biphasic, although the difference between infected and uninfected cells at 24 hpi was not significant (Figure 2A). Extracellular HMGB1 levels were significantly elevated at 6 and 24 hpi, similar to the biphasic pattern of increased cyto-HMGB1 and decreased nuclear-HMGB1 over the time course (Figure 2B). The increased extracellular HMGB1 at 6 hpi occurred in the absence of increased gene transcription at this time (Figure 2C), suggesting that the replenished nuclear HMGB1 at 12 hpi is likely a consequence of re-cycling from the cytoplasm, possibly through the modification of HMGB1’s two nuclear localization signals, or from the extracellular space through pathways such as macropinocytosis (Canton, 2018; Torriani et al., 2019). Using an anti-RSV antibody, RSV was not detected until 12 hpi, presumably due to the requirement for infection and then protein translation (Figure 2D). RSV gene expression escalated rapidly between 12 and 18 hpi (Figure 2E), prior to the peak of necroptosis at 24 hpi (Figures 1D–F). However, whereas < 25% of cells were RSV + at 24 hpi, almost twice that number were pRIPK1+ and pMLKL+, suggesting that endogenous RSV-induced factors likely contribute to the phosphorylation of necroptosis proteins and the associated “late” release of HMGB1.

### Respiratory Syncytial Virus Induces Necroptosis in Human Airway Epithelial Cells in an High Mobility Group Box 1-Dependent Manner

Given the early increase in extracellular HMGB1, we hypothesised that this contributes to the necroptosis-mediated release of “late” HMGB1. Neutralisation of HMGB1 using a neutralising antibody attenuated RSV-induced cell death (as determined by PI staining) and significantly decreased the phosphorylation of RIPK1, RIPK3, and MLKL at 24 hpi (Figures 3A,B). Anti-HMGB1 decreased the concentration of extracellular HMGB1, although an important caveat is the presence of the neutralising Ab in the culture medium (Figure 3C). In contrast, the number of cyto-HMGB1+ and RSV + cells was not affected by anti-HMGB1 (Figures 3C,D), highlighting a potential disconnect between cyto-HMGB1 expression and extracellular HMGB1 levels.

### Respiratory Syncytial Virus-Induced Necroptosis is Mediated *Via* a High Mobility Group Box 1/Receptor for Advanced Glycation Endproducts Axis

Necroptosis can be induced through the activation of various pattern recognition receptors, including RAGE (Upton et al., 2012; Gao et al., 2014; Schock et al., 2017; Amarante-Mendes et al., 2018; Faust et al., 2020). As HMGB1 is a ligand of RAGE, we hypothesised that the early release of HMGB1 activates RAGE, triggering necroptosis and late HMGB1 release. Consistent with this, pre-treatment of hAECs with the RAGE antagonist, FPS-ZM1 (Deane et al., 2012), significantly decreased pRIPK1, pRIPK3 and pMLKL expression, and PI + cells at 24 hpi, although no significant decrease was observed in dsDNA (Figures 4A,B). In contrast, no effect was observed at 6 hpi (Supplementary Figures S2A,B). Similar to the findings with anti-HMGB1, despite the significant decrease in extracellular HMGB1 levels, RAGE antagonism did not significantly lower the number of cyto-HMGB1+ AECs (Figure 4C). Nor was the number of RSV immunoreactive cells affected (Figure 4D). As expected, inhibition of RAGE signalling at 6 h did not affect extracellular HMGB1 levels at 6 hpi (Supplementary Figure S2C). Taken together these findings suggest that the release of “early” HMGB1 acts on RAGE to induce necroptosis at 24 hpi, leading to the subsequent release of “late” HMGB1.

### Exogenous High Mobility Group Box 1 and Caspase Inhibition Induces Necroptosis in Human Airway Epithelial Cells

Dual treatment with TNF-α and the pan-caspase inhibitor vZAD is an established method to induce necroptosis (Vanlangenakker et al., 2011; Cai et al., 2014). To further implicate HMGB1 as an inducer of necroptosis, we treated hAECs with recombinant HMGB1 and vZAD in the absence or presence of FPS-ZM1. At 24 hpi, HMGB1/vZAD treatment significantly increased cell death markers (Figure 5A) and the fraction of pRIP1K, pRIP3K and pMLKL immunoreactive hAECs (Figure 5B). As with RSV-associated necroptosis, HMGB1/vZAD-induced necroptosis was attenuated in the presence of the RAGE antagonist, FPS-ZM1, demonstrating that HMGB1/RAGE signalling induces necroptosis in hAECs (Figure 5). Of interest, recombinant HMGB1 alone increased pRIPK1, pRIPK3 and pMLKL expression, however, unlike dual HMGB1/vZAD treatment, this was not associated with cell death. Together, these data suggest that HMGB1 can act as a priming signal, promoting the phosphorylation of RIPK1/3 and MLKL, and that this may promote later cell death in response to additional signals from the microenvironment.

### pMLKL Inhibition Decreases Early High Mobility Group Box 1 Release Induced by Respiratory Syncytial Virus

As an early (6 hpi) increase in RSV-associated RIPK1, RIPK3, and MLKL phosphorylation was observed in hAECs in the absence of cell death (Figure 1), we next sought to determine whether the necroptosis-associated kinases influence the early, non-cell death dependent release of HMGB1. Treatment of the hAECs with a RIPK1 inhibitor (GSK’963) or a pRIPK3 inhibitor (GSK’872), did not prevent the RSV-induced fall in the fraction of nuclear HMGB1+ cells, however both inhibitors markedly attenuated the increase in cyto-HMGB1+ cells (Figures 6A,B). In contrast, the MLKL inhibitor ablated the loss of nuclear HMGB1 but had no effect on the RSV-induced increase in cyto-HMGB1 expression (Figure 6C). Inhibition of RIPK1 or MLKL, but not RIPK3, led to a significant decrease in extracellular HMGB1 levels at 6 hpi (Figures 6A–C). Taken together, these data suggest that the early release of HMGB1 in response to RSV infection is preferentially mediated through the activation of pRIPK1 and pMLKL.

## Discussion

RSV infection of hAECs induces the release of HMGB1 at 24 hpi in a necroptosis dependent manner (Simpson et al., 2020). In this study, we identified that RSV infection induces the phosphorylation of RIPK1, RIPK3 and MLKL and accompanying release of HMGB1 much earlier at 6 hpi. Significantly, MLKL inhibition attenuated both the early (6 hpi) and late (24 hpi) release of HMGB1, however, only the late phase was associated with cell death, suggesting that MLKL contributes to other cellular processes that mediate HMGB1 release independently of cell death. Additionally, we identified a feed-forward amplification loop whereby the early HMGB1 release promotes late HMGB1 release following the activation of RAGE-initiated necroptotic cell death.

By performing extensive time course analyses, we identified that RSV infection of hAECs leads to an early (6 hpi) and late (24 hpi) increase in RSV-induced RIPK1/RIPK3/MLKL phosphorylation and corresponding HMGB1 release. This finding is reminiscent of other studies that have shown early RIPK1/RIPK3/MLKL phosphorylation (e.g., in response to LPS or TNF-α stimulation) can occur much earlier than the induction of cell death, and that this signalling event appears to promote the production of pro-inflammatory cytokines (Najjar et al., 2016; Zhu P. et al., 2018). We hypothesised that the early release of HMGB1 acts in a paracrine manner to amplify the release of HMGB1, and, that this is mediated through an HMGB1/RAGE axis that induces necroptosis. Consistent with this, treatment with a RAGE antagonist decreased the fraction of pRIPK1, pRIPK3, pMLKL and PI positive hAECs, and lowered the level of extracellular HMGB1 at 24 hpi. Identical findings were observed in RSV infected hAECs when the levels of early HMGB1 in the supernatant were neutralised with anti-HMGB1. Further implicating HMGB1/RAGE signalling as an effective inducer of necroptosis in hAECs, we found that treating naïve hAECs with exogenous HMGB1 and a pan-caspase inhibitor (to block apoptosis) was sufficient to increase pRIPK1, pRIPK3, pMLKL expression and induce cell death. Moreover, in this setting, the induced phosphorylation of RIPK1 and RIPK3 was attenuated in the presence of the RAGE antagonist, supporting the notion that activation of RAGE in particular microenvironments can promote necroptosis. Of note, RAGE has been shown to directly interact with RIPK3 in endothelial cells (Faust et al., 2020), supporting our findings. However, we cannot exclude the possibility that the HMGB1/RAGE pathway induces a downstream mediator, such as TNF-a, that then initiates necroptosis. We have shown previously that minimal caspase-3 activity or active caspase-3 expression is observed following RSV infection of hAECs, in contrast to infection with other respiratory viruses (Baturcam et al., 2017; Simpson et al., 2020). Mechanistically, this appears to occur via the activation of NF-kB and the upregulation of BCl-1 and other caspase-suppressive proteins (Thomas et al., 2002; Bitko et al., 2007). Therefore, we assessed exogenous HMGB1 with a pan-caspase inhibitor to simulate the *in vivo* environment during an RSV infection. HMGB1 treatment alone increased the expression of pRIP1K, pRIPK3, pMLKL expression, which is consistent with a role for these kinases in promoting exosome release, and pro-inflammatory outcomes (e.g., cytokine responses) (Negroni et al., 2017; Yoon et al., 2017; Zhu K. et al., 2018). However, HMGB1 did not affect cell viability, highlighting the requirement of a second signal to trigger cell death. Intriguingly, we previously demonstrated in a neonatal mouse model that severe viral bronchiolitis is characterised by AEC necroptosis (Simpson et al., 2020) and, in the same mouse model, that the antagonism of RAGE attenuates the inflammatory response, lowers HMGB1 levels in the bronchoalveolar lavage and prevents the onset of type-2 inflammation and airway remodelling (Loh et al., 2020), although it remains to be determined whether this intervention decreased the extent of the necroptotic cell death.

The levels of extracellular HMGB1 were similar between the early and the late phases, however early HMGB1 release occurred in the absence of an increase in PI and sytox green staining and was not associated with an increase in extracellular dsDNA levels, implicating a cell death-independent process. While we did not evaluate HMGB1 immunoreactivity and cell death stains in the same cells, ∼10% of all hAECs were Sytox green positive at 6 hpi, whereas ∼40% of all cells at this time exhibited HMGB1 in the cytoplasm. Therefore, the vast majority of cells with cytoplasmic HMGB1 were not dying. Interestingly, the early phase HMGB1 release was attenuated by the MLKL inhibitor, NSA. The apparent relationship between MLKL and HMGB1 was also independent of cell death, as only 20% of the pMLKL positive hAECs were positive for Sytox green at 6 hpi. A recent investigation using various immortal cells lines identified that MLKL contributes to the transport of cellular proteins in addition to regulating necroptosis. Specifically, MLKL was shown to be required for the effective generation of multi-vesicular bodies, which fuse with lysosomes or the plasma membrane to create extracellular vesicles (Yoon et al., 2017). Of interest, HMGB1 has been shown to be released from activated monocytes via non-classical vesicle secretion (Gardella et al., 2002). This concept may partly explain the lack of concordance between cytoplasmic HMGB1 and extracellular HMGB1. Indeed, the loss of nuclear HMGB1 more closely associated with extracellular HMGB1, suggesting that nuclear HMGB1 exits *via* an additional pathway. Notably, inhibition of MLKL was particularly effective (in comparison to inhibition of RIPK1 or RIPK3) at preventing the early release of HMGB1, and this effect appeared to be independent of the RSV-induced increase in cytoplasmic HMGB1 levels. A limitation of our study was the semi-quantitative analysis of HMGB1 expression in the cells. Future studies should seek to employ advanced microscopy techniques to quantify the expression of HMGB1 in each cellular compartment and to trace its intracellular and intercellular movement. At the intracellular level, HMGB1 has been located in the nucleus, cytoplasm, mitochondria and vesicles (Gardella et al., 2002; Tian et al., 2007; Ibrahim et al., 2013; Hyun et al., 2016; Deng et al., 2019). At the extracellular level, HMGB1 has been shown to bind various molecular moieties (e.g., ssDNA, ssRNA, LPS, IL-1β, nucleosomes) and can support their internalisation through RAGE (Sha et al., 2008; Urbonaviciute et al., 2008; Bianchi, 2009; Bertheloot et al., 2016). Hence, as an alarmin, HMGB1 may be expelled in response to “danger” (infection, injury, stress) in order to sample the external environment, with this HMGB1-bound material then ingested to initiate intracellular recognition and guide the cellular response (and host defence). Further studies are needed to explore the mechanism of early HMGB1 release and specifically whether this is mediated *via* an MLKL-exosome pathway.

The biphasic pattern of phosphorylated RIPK1, RIPK3 and MLKL expression and release of HMGB1 after RSV infection may be in part mediated by the lifecycle of RSV. RSV protein translation in host cells is typically detectable from 6 hpi (Bakre et al., 2015), suggesting that the early induction of RIPK1, RIPK3 and MLKL phosphorylation occurs in response to detection of viral pathogen-associated molecular patterns by pattern recognition receptors (Spann et al., 2014; Brault et al., 2018). These typically elicit a rapid type I IFN response, which is also known to induce the phosphorylation of RIPK1 and RIPK3 (Thapa et al., 2013; Brault et al., 2018). Once RSV proteins are expressed, these may suppress apoptosis as discussed, and additionally, other RSV proteins such as M protein may contribute to HMGB1 trafficking and the phosphorylation of necroptosis proteins, as, for example, RSV-M was found to mediate large scale changes in cellular physiology, such as cell cycle arrest, in hBECs (Bian et al., 2012). We did not extensively explore the effect of inhibiting necroptosis on infectious RSV, as the primary focus of this study was on the early release of HMGB1. However, we have previously found that inhibition of necroptosis reduces the number of RSV + cells (Simpson et al., 2020), suggesting that necroptosis helps to facilitate the release and spread of RSV.

In conclusion, RSV infection induces the release of HMGB1 and the phosphorylation of RIPK1, RIPK3 and MLKL in a biphasic manner. The early release of HMGB1 acts in a paracrine manner signalling via RAGE to initiate the late release of HMGB1. Of note, whereas the late phase is associated with necroptosis, the early phase occurs in the absence of cell death. Despite this, the release of early HMGB1 is attenuated by the MLKL inhibitor NSA, suggesting that MLKL contributes to the release of HMGB1 via a non-cell death process. Further studies are needed to determine whether MLKL is a tractable therapeutic target to reduce the severity of viral bronchiolitis, irrespective of whether epithelial cell death contributes to the underlying endotype driving disease pathogenesis.

## Data Availability

The raw data supporting the conclusions of this article will be made available by the authors, without undue reservation.

## Supplementary Material

Supplementary Figure S1 Cell death and expression of necroptosis proteins after RSV infection. hAECs were infected with RSV (MOI of 1) and endpoints were assessed at 0, 2, 6, 12, 18, 24, and 48 h post infection. **(A)** Representative images (magnification ×40) of Sytox Green, cleaved caspase-3 (CC3), pRIP1K, pRIPK3, and pMLKL. **(B)** Representative images (magnification ×40) of Sytox green and pMLKL positive hAECs, and quantification of Sytox green positive cells. The data are presented as mean ± SEM. The data were analyzed using a two-way ANOVA and Sidhak’s multiple comparisons test. Each data point represents 6 donors. * *p* < 0.05, ***p* < 0.01, denotes significance between infected and uninfected cells at the specific time point.

Supplementary Figure S2 RAGE mediates RSV and HMGB1-dependent cell death at 24 hpi, but not 6 hpi. hAECs were pre-treated with/without a RAGE antagonist and infected with RSV (MOI of 1) Endpoints were assessed at 6 hours post infection. **(A)** Quantification of pRIPK1 positive hAECs. **(B)** Quantification of pMLKL positive hAECs. **(C)** HMGB1 expression in supernatant. Data are presented as dot plots with each data point representing one of 5 donors. The data were analyzed using a paired T-test.
