# Supplementary figures and images for: MLKL Regulates Rapid Cell Death-independent HMGB1 Release in RSV Infected Airway Epithelial Cells
Source: Front Cell Dev Biol. 2022 May 31;10:890389. doi: 10.3389/fcell.2022.890389 (PMC9194532; doi:10.3389/fcell.2022.890389)

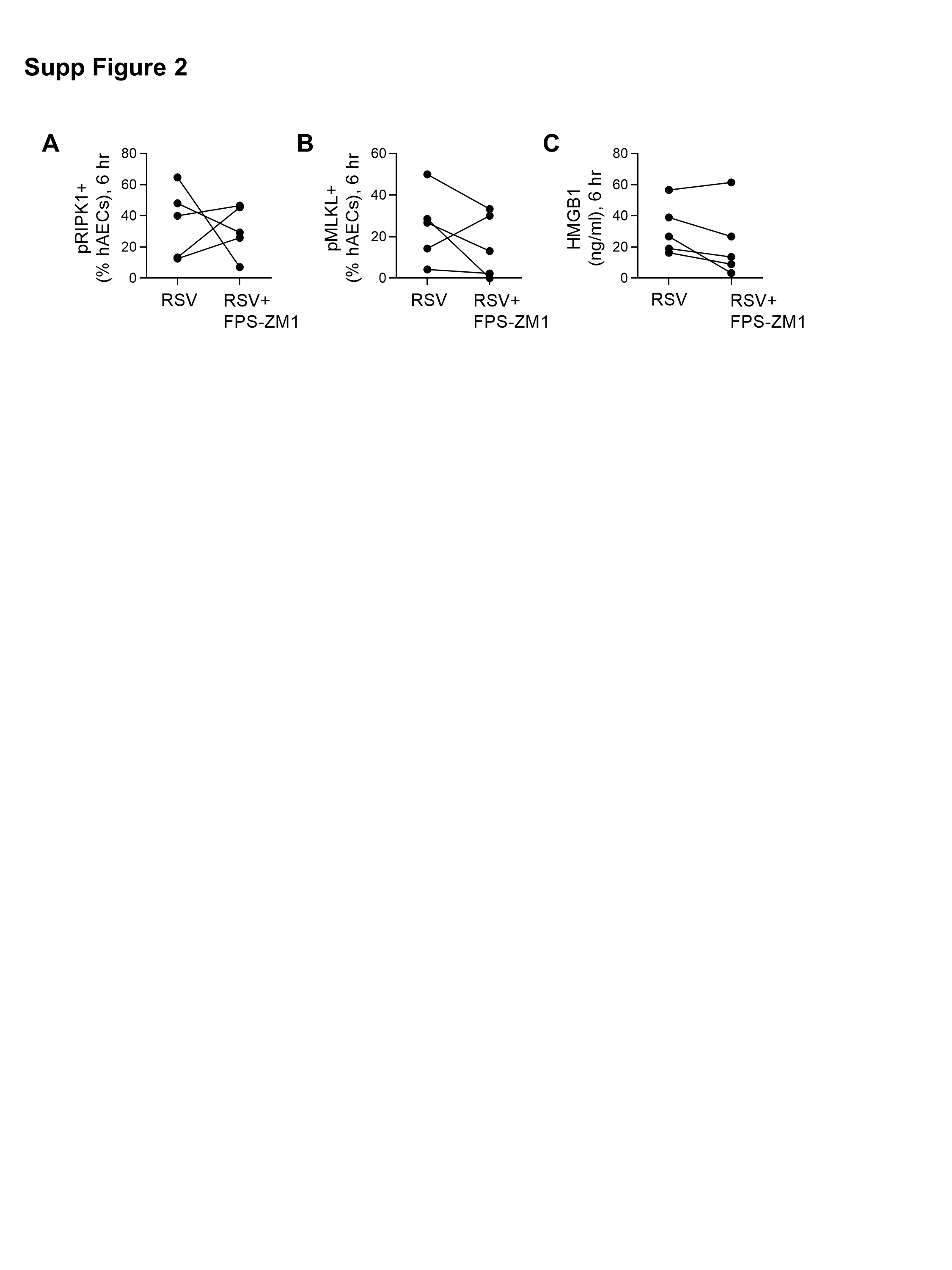

Supplement: Supplementary file 1 [file Image2.TIF]

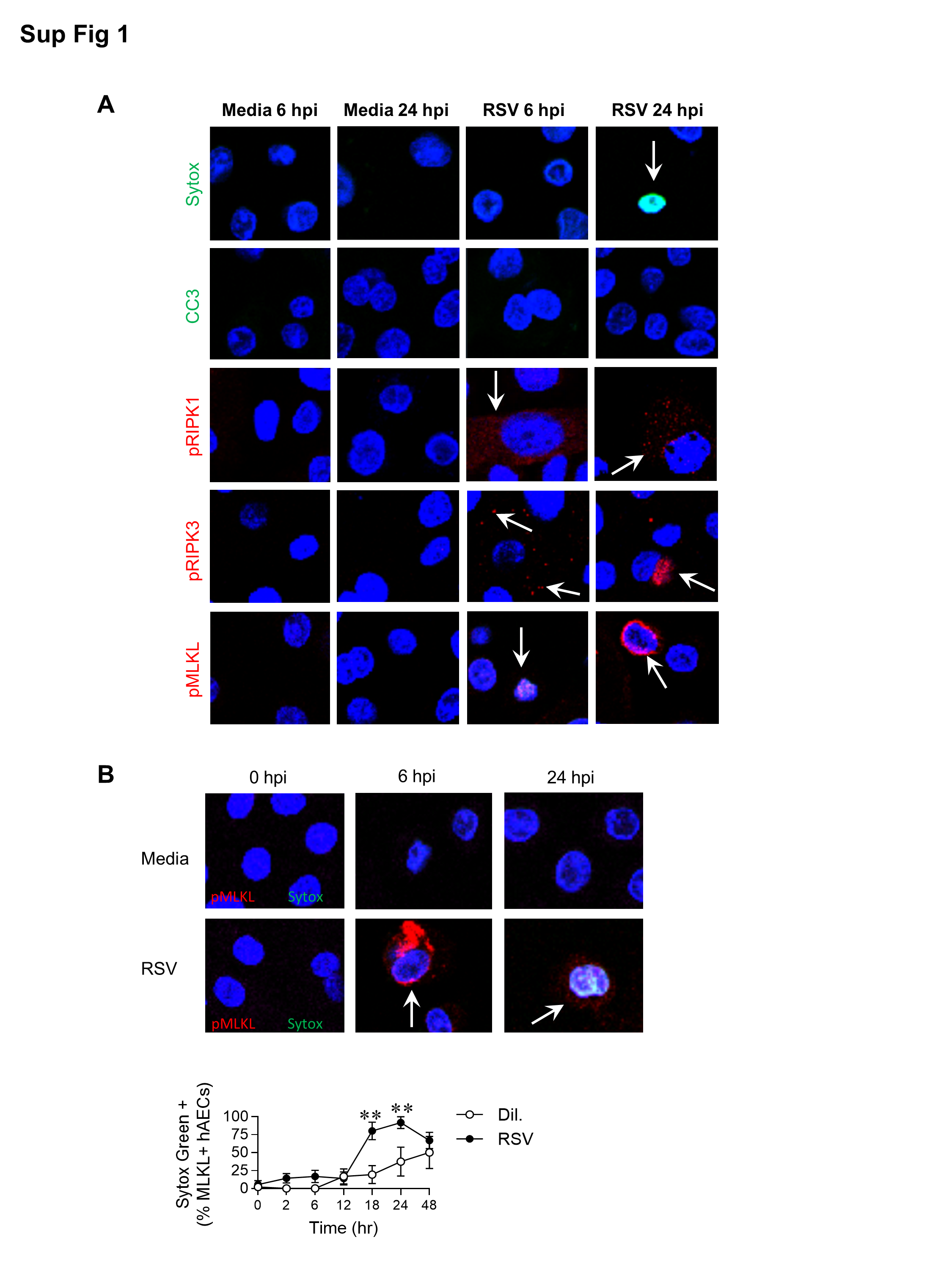

Supplement: Supplementary file 2 [file Image1.TIF]
